# Physicochemical, Bioactive and Technofunctional Properties of Instant Black Mashua Flour Processed by Extrusion and Drum Drying

**DOI:** 10.3390/foods15142486

**Published:** 2026-07-14

**Authors:** Alejandro Coloma, Edith Galindo-Quispe, Edgar Gallegos Rojas, Herbert Callo, Cynthia Castillo Santos, Maria Mamani-Mamani, Justo Gallegos Rojas, Arturo Zaira-Churata, Jorge Apaza-Cruz, Nancy Curasi Rafael, Cristina Valencia-Sullca, Ulises Alvarado

**Affiliations:** 1 Escuela Profesional de Ingeniería Agroindustrial, Facultad de Ciencias Agrarias, Universidad Nacional del Altiplano, Av. Floral 1153, Puno 21001, Peru; acoloma@unap.edu.pe (A.C.); edi970160@hotmail.com (E.G.-Q.); edgallegosr@unap.edu.pe (E.G.R.); herbert.callo@unap.edu.pe (H.C.); cynthiacastillo@unap.edu.pe (C.C.S.); maria.mamani@unap.edu.pe (M.M.-M.); sipia50@gmail.com (J.G.R.); 2 Instituto de Investigacion e Innovación en Producción, Seguridad Alimentaria y Agroindustria—IPSAA, Facultad de Ciencias Agrarias, Universidad Nacional del Altiplano, Av. Floral 1153, Puno 21001, Peru; ncurasi@upeu.edu.pe; 3 Escuela Profesional de Nutrición Humana, Facultad de Ciencias de la Salud, Universidad Nacional del Altiplano, Av. Floral 1153, Puno 21001, Peru; azaira@unap.edu.pe; 4 Escuela Profesional de Ingeniería Electrónica, Universidad Nacional del Altiplano, Av. Floral 1153, Puno 21001, Peru; jlapaza@unap.edu.pe; 5 Escuela Profesional de Ingeniería Ambiental, Facultad de Ingeniería y Arquitectura, Universidad Peruana Unión, Carretera Arequipa Km 6, Juliaca 21100, Peru; 6 Institute of Chemistry and Biology of Membranes and Nano-Objects (CBMN), Unité Mixte de Recherche (UMR) 5248, Centre National de la Recherche Scientifique (CNRS), University of Bordeaux, Bordeaux Institute of Technology (Bordeaux INP), European Institute of Chemistry and Biology (IECB), 2 Allée Geoffroy Saint-Hilaire, 33600 Pessac, France; cristina.valencia-sullca@u-bordeaux.fr

**Keywords:** bioactive compounds, black mashua, extrusion, drum drying, technofunctional properties

## Abstract

Black mashua (*Tropaeolum tuberosum*), also known as purple mashua, is an Andean plant with high nutritional and medicinal value. It has traditionally been used to treat prostate conditions. This study examined how the agroecological origin (Puno and Junín) and processing methods (extrusion and drum drying) affected the physicochemical, bioactive, and technofunctional properties of instant black mashua flour. Both factors were found to significantly influence the content of bioactive compounds and technofunctional behaviour of the flour. Although extrusion increased the content of total phenols, flavonoids, carotenoids, and total glucosinolates, it decreased the levels of vitamin C and anthocyanins, likely due to their thermal sensitivity. From a technological perspective, extrusion also resulted in higher bulk density, water absorption capacity, and swelling power. In contrast, drum drying produced higher water solubility and gelatinisation index values. Samples from Puno generally exhibited higher glucosinolate contents than those from Junín, while extrusion proved more effective in enhancing glucosinolate retention and improving functional properties. These findings suggest that the processing method should be chosen based on the specific bioactive compounds and technological functionalities that are prioritised in each agroecological zone.

## 1. Introduction

*Tropaeolum tuberosum*, known as mashua in Peru and Ecuador, isaño in Bolivia, and cubio in Colombia, is an Andean tuber that has been cultivated and domesticated since pre-Columbian times. It remains an important food source and a valuable crop for many rural families in the Andean region today [1]. Mashua has significant nutritional value as it contains proteins, carbohydrates, dietary fibre, β-carotene, vitamin C, calcium, potassium, phosphorus, iron and zinc. Furthermore, it contains all the essential amino acids except tryptophan [2,3]. As well as being important for nutrition, mashua has been widely used in traditional medicine to treat various ailments, including pulmonary, dermatological, venereal, renal and prostate disorders [4,5,6,7,8]. Its pharmacological relevance is attributed to its high concentration of bioactive compounds, including polyphenols, anthocyanins, carotenoids, glucosinolates, alkaloids, flavonoids, tannins, hydroxybenzoic acids, isothiocyanates and phytosterols. These compounds exhibit antioxidant activity and potential anti-tumour effects [4,9,10]. Mashua tubers display a wide range of colours, including yellow, white and purple. Among these, the purple genotype has been reported to exhibit higher levels of total anthocyanins, flavonoids, phenolic compounds, tannins and antioxidant activity [11].

Despite its nutritional and functional potential, the utilisation of black mashua is limited, primarily because of the lack of research into its processing and technological applications [12]. It is well known that food processing can significantly affect the physicochemical characteristics and stability of bioactive compounds. Several studies have demonstrated that thermal treatments such as blanching, pasteurisation, sterilisation and roasting can reduce the concentration of antioxidant bioactive compounds [13]. Similarly, Sharma et al. [14] reported that thermal processing alters the bioactive constituents, antioxidant capacity and functional properties of quinoa grains. Ismail et al. [15] also found that conventional heat treatments negatively impact the quality of fruit pulp, particularly the levels of ascorbic acid and other heat-sensitive bioactive compounds.

Adding value to purple mashua is essential for the sustainability of the Andean communities that cultivate it. One effective strategy for extending its shelf life and facilitating its incorporation into food formulations is to transform it into instant flour. This is because fresh mashua tubers have a high moisture content (90.84 ± 0.56%), which limits their preservation and storage stability [16]. Instant flours can be produced using various technologies, such as drum drying, spray drying and extrusion. These processes all involve heat-induced starch pregelatinisation [17,18]. Extrusion is a technology that uses high temperatures, pressure, and shear forces to modify the structure of food and improve its functional properties [19]. In contrast, drum drying involves cooking the raw material in water or steam, then drying it on heated drums before milling to produce a fine powder [20].

A comprehensive understanding of the composition of mashua and how its bioactive compounds are affected by processing is crucial for its industrial development and commercialisation [13]. Although previous studies have characterised the nutritional composition and bioactive compounds of mashua tubers from different genotypes and agroecological regions [3,11], information regarding the effects of processing technologies on the bioactive compounds, glucosinolate profile, and technofunctional properties of black mashua remains limited. In particular, comparative studies evaluating extrusion and drum drying as technologies for producing instant black mashua flour are scarce. The objective of this study was therefore to evaluate the influence of different processing methods for producing instant black mashua flour from two Peruvian agroecological zones on its physicochemical, bioactive and technofunctional properties.

## 2. Materials and Methods

### 2.1. Materials

Black mashua tubers were cultivated in two agroecological zones of Peru: Junín (3384 m a.s.l., latitude 11°46′45.89″ S and longitude 75°29′45.00″ W) and Puno (3880 m a.s.l., latitude 15°38′30″ S and longitude 69°49′50″ W). After harvest, the tubers were transported to the laboratory, washed to remove surface impurities and stored at 25 ± 2 °C under a relative humidity of 68.1 ± 1.5% until further processing.

### 2.2. Processes for Obtaining Instant Mashua Flour

#### 2.2.1. Extrusion Process

Instant black mashua flour was produced by extrusion, based on the methodology outlined by Paucar-Menacho et al. [10], with some modifications. Fresh mashua tubers were weighed, washed with water, then disinfected using a 50 ppm sodium hypochlorite solution. The tubers were sliced manually with a stainless-steel knife into discs approximately 2 mm thick, then dehydrated in a tray dryer until they reached a moisture content of 10%. These slices were then milled using a hammer mill (Vulcano model MV 35–45 with a 7.5 HP motor) to produce flour with a particle size of 2.0 mm. The mashua flour was conditioned with distilled water until it reached a moisture content of 23%, after which it was extruded using a twin-screw extruder (Inbramaq, model Labor PQ DRX-50, Ribeirão Preto, Brazil). This extruder was equipped with seven heating zones, a screw length of 870 mm, a screw diameter of 32 mm, and a die with an orifice diameter of 6 mm. The barrel zones were set to temperatures of 30, 40, 50, 70, 90, 120 and 150 °C, respectively. The screw speed was maintained at 305 rpm with a feed rate of 6 kg/h. The expanded extrudates were discharged through the die and cut using a rotary knife operating at 15 Hz. After extrusion, the samples were cooled to room temperature, packaged in biaxially oriented polypropylene bags, and stored in the dark until analysis.

#### 2.2.2. Drum Drying Process

The fresh mashua tubers were weighed, washed with drinking water and disinfected using a solution of sodium hypochlorite at 50 ppm. They were then cooked in water at 95 °C for 25 min, crushed and homogenised to produce a uniform pulp. The pulp was then dried using a double-drum rotary dryer (Buflovak, New York, NY, USA), which was operated at a saturated steam pressure of (552 kPa) 80 psi, a drum surface temperature of 130 °C, and a rotational speed of 0.27 rpm. The resulting dried mashua flakes were milled to produce a fine flour, packaged in polyethylene bags and stored in a dark place until further analysis.

### 2.3. Proximate Composition Analysis

Proximate analysis of black mashua flour samples was carried out according to standard AOAC procedures [21]. The moisture content was determined using the gravimetric method (AOAC 925.09), the crude protein content by the Kjeldahl method (AOAC 954.01), the lipid content by Soxhlet extraction using hexane as the solvent (AOAC 920.39), the ash content gravimetrically (AOAC 923.03) and the crude fibre content by the acid–alkali hydrolysis method (AOAC 962.09). Total carbohydrate content was calculated by difference on a dry weight basis by subtracting the sum of the protein, lipid, ash, and crude fibre contents from 100. The results are expressed as g/100 g of dry weight (DW).

### 2.4. Analysis of Bioactive Compounds

#### 2.4.1. Total Anthocyanin Content (TAC)

The TAC of mashua extracts was determined using the pH differential method (AOAC, 2006). Absorbance was measured at 520 and 700 nm using buffer solutions at pH 1.0 and 4.5. For anthocyanin quantification, a molar extinction coefficient of 26,900 L·cm^−1^·mol^−1^ and a molecular weight of 449.2 g/mol were used, assuming cyanidin-3-glucoside as the reference compound [3]. The results were expressed as milligrams of cyanidin-3-glucoside equivalents (CGE) per gram of dry matter (mg CGE/g DM).

#### 2.4.2. Total Flavonoid Content (TFC)

The TFC of the black mashua instant flour extracts was determined using the aluminium chloride colorimetric method described by Chang et al. [22], with slight modifications. Quercetin was used as the standard for the calibration curve. Ten milligrams of quercetin was dissolved in 80% ethanol, and standard solutions were prepared at concentrations of 2.5, 5.0, 7.5, and 10.0 µg/mL. The diluted standard solutions (500 µL) were mixed separately with 1.5 mL of 95% ethanol, 100 µL of 2% aluminium chloride, 100 µL of 1 M potassium acetate, and 4.5 mL of distilled water. After being incubated at room temperature (25.5 ± 1 °C) for 30 min, the absorbance of the reaction mixture was measured at a wavelength of 415 nm using a spectrophotometer (Genesys 20, Thermo Scientific, Mississauga, ON, Canada). For the blank sample, aluminium chloride was replaced with distilled water. Similarly, 500 µL of the ethanolic extracts was processed with aluminium chloride under the same conditions to determine their flavonoid content. The results were expressed as milligrams of quercetin equivalents (QE) per gram of dry matter (mg QE/g DM).

#### 2.4.3. Total Phenolic Content (TPC)

The TPC was determined using the Folin–Ciocalteu method as described by Chirinos et al. [23]. For this purpose, 50 µL of the extract was mixed with 100 µL of 0.2 N Folin–Ciocalteu reagent (Sigma-Aldrich Chemie, Steinheim, Germany) and left to react for 5 min. Subsequently, 200 µL of a 20% sodium carbonate (Na_2_CO_3_) solution (Labosi, Paris, France) was added to the mixture, which was then homogenised and brought up to a final volume of 1700 µL with distilled water. The mixture was then incubated at room temperature for 30 min, and the absorbance was measured at 765 nm against a 70% ethanol blank using a spectrophotometer (Genesys 20, Thermo Scientific, Mississauga, ON, Canada). A calibration curve was constructed using gallic acid (Sigma-Aldrich Chemie, Steinheim, Germany) at concentrations of 0–10 µg/mL in 70% ethanol. Determinations were performed in triplicate and the total phenolic content was expressed as milligrams of gallic acid equivalents (GAE)/g of dry matter (DM).

#### 2.4.4. Antioxidant Capacity (DPPH Assay)

The previously obtained extracts were evaluated for antioxidant activity using the DPPH (2,2-diphenyl-1-picrylhydrazyl) radical scavenging method [23]. For this purpose, 50 µL of each extract was mixed with 1.5 mL of a methanolic DPPH solution (100 mM). The mixture was shaken vigorously and left to stand at room temperature for 30 min. The absorbance was then measured at 517 nm using a spectrophotometer (Genesys 20, Thermo Scientific, Mississauga, ON, Canada). Antioxidant activity was determined using a standard curve obtained with an ethanolic solution of Trolox (6-hydroxy-2,5,7,8-tetramethylchroman-2-carboxylic acid) at different concentrations. The results were expressed as µmol of Trolox equivalents (TE) per gram of dry matter (DM).

#### 2.4.5. Total Carotenoid Content

Carotenoids were extracted following the procedure described by Coloma et al. [3]. Quantification was carried out by high-performance liquid chromatography (HPLC) using an Agilent Technologies 1200 Series system (Agilent Technologies, Palo Alto, CA, USA) equipped with a diode array detector (DAD) and operated with ChemStation V03.02 software. Chromatographic separation was performed on a Zorbax Eclipse SB-C18 analytical column (4.6 mm × 75 mm, 3.5 µm; Agilent Technologies, Palo Alto, CA, USA), coupled to a Zorbax Eclipse XDB-C18 guard column (4.6 mm × 12.5 mm, 3.5 µm; Agilent Technologies, Palo Alto, CA, USA) to protect the stationary phase. The system operated under isocratic conditions using a mobile phase consisting of acetonitrile (40%) and 2-propanol (60%) at a flow rate of 1.0 mL/min. The column temperature was maintained at 25 °C, and the injection volume was 1 µL. Detection was performed at 450 nm. The total run time was 10 min. Carotenoid quantification was based on peak areas and calculated using calibration curves constructed with β-carotene standards. Results were expressed as milligrams per gram of dry matter (mg/g DM).

#### 2.4.6. Vitamin C

Vitamin C was extracted from instant mashua flour using the method outlined by Coloma et al. [3]. Quantification was performed using high-performance liquid chromatography (HPLC) with an Agilent Technologies 1200 Series system (Agilent Technologies, Palo Alto, CA, USA), which was equipped with a diode array detector (DAD) and operated with ChemStation V03.02 software. Chromatographic separation was achieved using a Zorbax Eclipse XDB-C18 column (4.6 mm × 75 mm, 3.5 µm) coupled with a Zorbax Eclipse XDB-C18 guard column (4.6 mm × 12.5 mm, 5 µm). An isocratic elution system was employed, using a mobile phase of 0.01 M KH_2_PO_4_ adjusted to pH 2.6, at a flow rate of 0.5 mL/min. The injection volume was 1 µL and the column temperature was maintained at 30 °C. Detection was performed at 250 nm. Vitamin C content was quantified based on peak areas using external calibration curves constructed with ascorbic acid standards, and the results were expressed as milligrams per gram of dry matter (mg/g DM).

#### 2.4.7. Glucosinolate Content

To determine the loss of glucosinolates, the extraction method described by Aguilar-Gálvez et al. [9] was employed. Five grams of crushed mashua tuber was weighed and placed in a 50 mL tube, to which 10 mL of hot (75 °C) 70% methanol was added. The samples were then incubated in a water bath at 75 °C for 30 min. The mixture was then centrifuged at 1520× *g* for 10 min at 4 °C and the resulting supernatant was transferred to a new tube. The extraction process was then repeated using the same procedure on the precipitate. The two resulting solutions were combined and 2 mL of glucosinolate extract was added to a DEAE-activated Sephadex A-25 chromatography column (20 µM sodium acetate). The glucosinolates were then desulfated using sulfatase. Following an 18 h reaction period at room temperature, the desulfated glucosinolates were eluted with MilliQ water to a final volume of 5 mL. The solution was then concentrated at 40 °C for 150 min and stored at −20 °C prior to high-performance liquid chromatography (HPLC) analysis. The same procedure was applied to samples obtained from both drying processes (extrusion and drum drying) to determine the percentage loss of glucosinolates.

### 2.5. Technofunctional Properties

#### 2.5.1. Bulk Density

Bulk density (BD) was determined using the method described by Otondi et al. [24]. Four grams of each sample were weighed into a 10 mL graduated cylinder. The samples were then compacted by tapping the cylinder gently ten times on a laboratory bench from a height of 5 cm. The final sample volume was recorded and bulk density was calculated using Equation (1). Although the tapping method involves a manual compaction step that may introduce some variability, it was selected because it is a widely adopted procedure in the characterisation of instant and extruded flours [25]. All measurements were performed in triplicate to minimise experimental error.

(1) $$Bulk\;density\left(\frac{g}{mL}\right)=\frac{Weight\;of\;the\;sample}{Volume\;of\;the\;sample\;after\;tapping}$$

#### 2.5.2. Water Absorption Index and Water Solubility Index

The method developed by Otondi et al. [24] for cereal products was adapted to determine the water absorption index (WAI) and the water solubility index (WSI). In brief, 2.5 g of the extrudate sample was ground and passed through a 500 µm sieve. The resulting powder was suspended in 12 mL of distilled water at 30 °C in a 15 mL centrifuge tube. The tube was shaken continuously for 30 min, after which the sample was centrifuged at 171.36× *g* for 10 min. The supernatant was carefully decanted into a pre-weighed beaker and dried in an oven at 90 °C until it reached a constant weight. The remaining gel was weighed, and the WAI was calculated from its mass using Equation (2). The amount of dry solids recovered from the supernatant was used to calculate the WSI, expressed as a percentage according to Equation (3).

(2) $$WAI=\frac{Weight\;of\;sediment}{Weight\;of\;dry\;solids}$$

(3) $$WSI\;(\%)=\frac{Weight\;of\;dissolved\;solids\;in\;supernatant}{Weight\;of\;dry\;solids}\times 100$$

#### 2.5.3. Swelling Power

The swelling power was determined using the method described by Otondi et al. [24]. One gram of the sample was weighed in triplicate and suspended in 20 mL of deionised water. The suspension was then heated in a water bath at 90 °C for 1 h with continuous stirring. Once cooled to 30 °C, the samples were centrifuged at 1200 rpm (417× *g*) for 10 min. The swollen granules were then decanted onto an aluminium plate and weighed. They were subsequently dried at 110 °C for 12 h and weighed again. Swelling power (Sp) was calculated using Equation (4).

(4) $$Sp=\frac{Weight\;of\;Sediment}{Weight\;of\;dry\;solids-Weight\;of\;dissolved\;solids\;in\;supernatant}$$

#### 2.5.4. Degree of Gelatinisation

The degree of gelatinisation of instant black mashua flour was determined based on the formation of a blue iodine–amylose complex released during starch gelatinisation following the method described by Huamani-Huamani et al. [25]. The samples were ground and sieved through an ASTM No. 80 mesh (particle size of 180 μm). A 50 mg sample was mixed with 50 mL of 0.05 M KOH solution and centrifuged at 4000 rpm for 10 min using a microprocessor-controlled centrifuge (model Q222TM). Aliquots of the supernatant (1 mL) were mixed with 1 mL of 0.05 M HCl and diluted to a final volume of 10 mL with deionised water. Then, 0.1 mL of iodine reagent (1 g of iodine and 4 g of potassium iodide dissolved in 100 mL of deionised water) was added. After thorough mixing, the absorbance was measured at 600 nm using a UV–Vis spectrophotometer (Jenway 6850, Bibby Scientific Ltd., Stone, UK), against a reagent blank. For the control samples, 0.05 M KOH and 0.05 M HCl were replaced with 0.5 M KOH and 0.5 M HCl, respectively. The reference blank consisted of 10 mL of distilled water and 0.1 mL of iodine reagent. The degree of gelatinisation was calculated according to Equation (5).

(5) $$Degree\;of\;gelatinization\left(\%\right)=\left(\frac{A1}{A2}\right)\times 100$$

where A1 is the absorbance of the test group at 600 nm, and A2 is the absorbance of the control group.

### 2.6. Statistical Analysis

A completely randomised experimental design was employed. The analysed data were derived from technical replicates obtained from a single, homogeneous batch for each evaluated sample. The effects of agroecological origin and processing method, as well as their interaction, were evaluated using two-way analysis of variance (ANOVA). Mean comparisons were performed using Tukey’s multiple range test at a significance level of *p* < 0.05. All determinations were conducted in triplicate. Statistical analyses were performed using Statgraphics^®^ Centurion software (version 19.2.02).

## 3. Results and Discussion

### 3.1. Physicochemical Composition

Table 1 presents the proximate composition on a dry matter basis of instant black mashua flour obtained using two processing methods and cultivated in two agroecological zones. Fresh black mashua from Junín showed slightly higher moisture (71.70 vs. 68.90 g/100 g FM) and protein content (9.97 vs. 9.41 g/100 g DW) compared to Puno samples, while Puno material exhibited higher fibre content (4.01 vs. 3.43 g/100 g DW). Carbohydrate, fat, and ash contents were comparable between zones. The protein content observed in this study is consistent with that reported by Coloma et al. [3] for the same black mashua genotype, ranging from 7.41 to 11.72 g/100 g DW. However, the moisture, fat, ash, fibre and carbohydrate contents were lower than previously reported ranges (74.51–89.72 g/100 g DW, 4.40–4.70 g/100 g DW, 5.32–6.66 g/100 g DW, 5.78–6.36 g/100 g DW and 70.73–76.99 g/100 g DW, respectively). Overall, the proximate composition values obtained were comparable to those reported by Apaza et al. [7]. Variations in the chemical composition of mashua have been attributed to factors such as geographic location, genotype, plant maturity, and environmental growing conditions [24,26], which may also explain the differences observed between samples cultivated in Junín and Puno.

The moisture content of instant black mashua flours produced by extrusion and drum drying in the Junín agroecological zone was found to be 10.43 g/100 g FM and 7.35 g/100 g FM, respectively. For flours obtained from mashua grown in Puno, moisture contents of 9.70 g/100 g FM and 7.18 g/100 g FM were observed for extrusion and drum drying, respectively. These values indicate low moisture levels, which are desirable for shelf-stable food products. As expected, the moisture content of the instant flours was markedly lower than that of the fresh tubers for both processing methods and growing locations. Similar moisture levels have been reported for extruded banana flour powders [27]: 3.75% to 6.34%. During the extrusion of starchy materials, the combined effect of high temperature and pressure followed by a sudden drop in pressure at the die promotes water evaporation, resulting in a reduction in moisture content in the expanded product.

The protein content of instant black mashua flour obtained from mashua cultivated in Junín was 12.28 g/100 g DW for extrusion and 10.97 g/100 g DW for drum drying. Similarly, flours produced from mashua cultivated in Puno exhibited protein contents of 10.22 g/100 g DW for extrusion and 8.94 g/100 g DW for drum drying. These values were comparable to those of the fresh samples, indicating that neither processing method resulted in significant protein loss. Mashua is recognised as a nutritionally valuable tuber due to its relatively high protein content and favourable amino acid profile [3].

The fat, ash, fibre, and carbohydrate content of instant black mashua flours produced by extrusion and drum drying were similar to that of the fresh tubers. This suggests that neither processing method significantly altered the overall composition of the flours. These results support those of Menegassi et al. [26], who found that extrusion processing did not result in significant nutrient loss in amaranth-based products.

### 3.2. Bioactive Compounds

**Figure 1:**
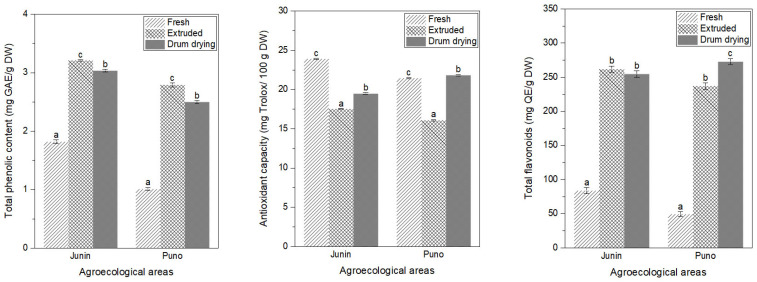

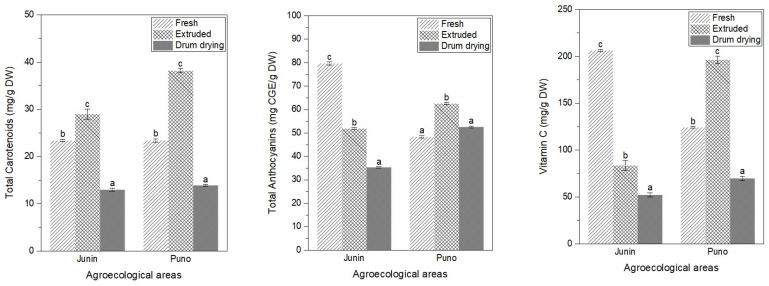
Bioactive compound content of instant black mashua flour (*Tropaeolum tuberosum*) obtained by extrusion and drum drying from two agroecological zones (Junín and Puno). Values are expressed as mean ± standard deviation (*n* = 3). Different lowercase letters indicate significant differences among treatments according to Tukey’s multiple comparison test (*p* < 0.05). GAE = gallic acid equivalents; QE = quercetin equivalents; CGE = cyanidin-3-glucoside equivalents; DW = dry weight.

Figure 1 present the bioactive compounds of instant black mashua flour obtained using two processing methods in the Junín and Puno agroecological zones.

#### 3.2.1. Total Phenolic Content (TPC)

Figure 1 shows the total phenolic content (TPC) of fresh and processed black mashua in the form of instant flour. The TPC of fresh black mashua grown in Junín and Puno was 1.82 ± 0.03 and 1.01 ± 0.03 mg GAE/g DW, respectively. These values are comparable to those reported by Coloma et al. [3] and Chirinos et al. [23]. The TPC of instant black mashua flour increased significantly (*p* < 0.05) in both agroecological zones after processing, by between 48% and 76% compared with the fresh samples. Similar behaviour was observed by Chávez et al. [28], who reported that extrusion can increase the concentration of bioactive compounds. This increase in TPC can be attributed to the disruption of the cell wall matrix caused by mechanical shear and thermal effects during extrusion, which favour the release of bound phenolic compounds. Furthermore, Sharma et al. [14] found that thermal processing, particularly dry heating, promotes the release of phenolic compounds and the formation of Maillard reaction products with phenolic and reductone-like structures. However, other studies have reported reductions in TPC after extrusion in crops such as pigmented maize, purple potatoes, yellow peas, barley and sorghum [29], indicating that the effect of extrusion depends strongly on the composition of the raw material and the processing conditions.

#### 3.2.2. Antioxidant Capacity (AC)

The antioxidant capacity of fresh black mashua grown in Junín and Puno was 24.30 ± 0.08 and 22.90 ± 0.08 mg Trolox/100 g DW, respectively. These values were lower than those reported by Chirinos et al. [23]. Processing into instant flour significantly reduced antioxidant capacity (*p* < 0.05) in both agroecological zones, with decreases of between 5% and 30% observed, particularly in extruded samples. This finding is consistent with that of Sanusi et al. [28], who reported that extrusion may reduce antioxidant activity depending on processing conditions and processing intensity.

#### 3.2.3. Total Flavonoid Content (TFC)

Extrusion processing markedly increased the total flavonoid content of instant black mashua flour. In samples from Junín, total flavonoid content (TFC) increased from 85.32 ± 4.31 to 261.96 ± 4.66 mg EQ/g DM, and in samples from Puno it increased from 52.81 ± 3.75 to 237.15 ± 4.65 mg EQ/g DM. These results are consistent with those reported by Sanusi et al. [28], who found that the flavonoid content of extruded products increases with higher moisture content, temperature, and screw speed. However, the effect of extrusion on flavonoids remains controversial, since Sharma et al. [14] reported the degradation of flavonoids at excessively high extrusion temperatures. Flavonoids are generally considered heat-sensitive compounds, and their stability depends strongly on processing parameters.

#### 3.2.4. Total Carotenoid Content (TCC)

The total carotenoid content of the samples varied between 12.99 ± 0.34 and 38.19 ± 0.44 mg/g DM (Figure 1). Carotenoid degradation was more pronounced in instant flours processed by drum drying; extrusion, on the other hand, resulted in an increase in carotenoid content. These values are considerably higher than those reported by Pacheco et al. [30] for yellow mashua (1.4 ± 0.1 mg/g DM). It has been widely reported that carotenoid content is reduced during drum drying, mainly because prolonged exposure to relatively high temperatures promotes oxidative degradation of carotenoids [31].

#### 3.2.5. Total Anthocyanin Content (TAC)

The total anthocyanin content (TAC) of fresh black mashua showed a marked geographical dependence, yielding 81.02 ± 0.66 and 51.64 ± 0.55 mg CGE/g DW for the Junín and Puno samples, respectively. These differences between agroecological zones may be related to environmental and agronomic factors, including cultivation altitude, since plants grown at higher altitudes typically synthesise greater amounts of photoprotective pigments in response to increased UV and UV-B radiation [32].

Processing significantly altered pigment stability; Junín samples exhibited high susceptibility, dropping to 51.91 mg CGE/g DW after extrusion and reaching their lowest level during rotary drum drying (35.35 mg CGE/g DW; *p* < 0.05). This reduction may be associated with the thermal sensitivity of anthocyanins and the additional hydrothermal pre-cooking step included in the drum-drying process, which could have promoted cumulative thermal degradation and leaching of these water-soluble pigments. Similar reductions in anthocyanin content after thermal processing have been reported in anthocyanin-rich foods [32].

In contrast, Puno samples exhibited a moderate increase in TAC after extrusion, which may reflect improved extractability rather than a true increase in native anthocyanin content during thermomechanical processing. Similar observations have been reported in extruded products, where processing conditions can influence both anthocyanin stability and extractability [33].

Overall, these results suggest that anthocyanin stability is influenced not only by processing conditions such as time and temperature, but also by characteristics associated with the raw material and agroecological origin. These findings highlight the importance of considering both cultivation environment and processing technology when developing value-added mashua-based products. This is consistent with recent studies that recognise mashua as a promising source of bioactive compounds and a valuable functional food ingredient [34].

#### 3.2.6. Vitamin C Content

The vitamin C content decreased substantially following extrusion processing, from 209.84 ± 1.02 mg/g DM to 83.63 ± 5.36 mg/g DM in the Junín samples. Conversely, the Puno samples exhibited an apparent increase in vitamin C content, increasing from 132.87 ± 1.03 to 196.59 ± 3.88 mg/g DM. These values are comparable to those reported by Pacheco et al. [30] for yellow isaño (82 ± 0.3 mg/g DM).

Ascorbic acid is highly unstable and sensitive to heat, light, and oxygen. Parveez Zia and Alibas [35] reported total ascorbic acid losses ranging from 79% to 87% during convective drying of cornelian cherries, confirming the susceptibility of this vitamin to thermal processing.

The variability observed between agroecological zones in the present study may be associated with differences in the initial vitamin C content and in the response of the plant matrix to processing conditions. The apparent increase observed in the Puno extrudate may reflect improved extractability and analytical recovery of ascorbic acid following thermomechanical processing rather than a true increase in vitamin C concentration. Similar effects of thermal processing on the measured concentration and recoverability of bioactive compounds have been reported previously [14]. However, this interpretation should be considered with caution, since the mechanisms involved were not directly evaluated in the present study.

When evaluating the alternative technology, it is important to note that the rotary drum drying process included a hydrothermal pre-cooking step (95 °C for 25 min) prior to drying. This prolonged exposure to elevated temperatures, together with the potential leaching of water-soluble compounds into the cooking medium, may have contributed to the marked reduction in vitamin C concentration observed in drum-dried samples. Similar effects of processing on the stability of bioactive compounds in mashua have been reported previously [36].

Overall, these results highlight the susceptibility of vitamin C to thermal processing and the importance of optimising processing conditions to better preserve this highly thermolabile compound.

#### 3.2.7. Glucosinolate Content

The glucosinolate (GLS) content of fresh and processed instant black mashua flour from different agroecological zones is presented in Table 2. The main glucosinolates identified were 4-methoxybenzyl glucosinolate (glucoaubrietin), benzyl glucosinolate (glucotropaeolin), and two positional isomers of hydroxybenzyl glucosinolate, namely *p*-hydroxybenzyl glucosinolate (glucosinalbin) and m-hydroxybenzyl glucosinolate (glucolepigramin).

The total GLS content of instant black mashua flour increased significantly (*p* < 0.05) after extrusion in both agroecological zones compared with the fresh material. In contrast, drum drying reduced total glucosinolate content in the Junín samples but increased it in the Puno samples. Thermal processing may facilitate glucosinolate extraction through tissue softening and cellular disruption, thereby improving analytical recovery. Campos et al. [36] reported considerable variability in glucosinolate content among mashua cultivars, with purple varieties exhibiting the highest concentrations. In their study, glucoaubrietin represented more than 96% of total glucosinolates and showed high thermal stability during common cooking treatments, including boiling, microwaving and baking. The predominance of aromatic glucosinolates, particularly glucoaubrietin, may contribute to the relatively high stability of glucosinolates during processing.

Marked differences were observed in glucosinalbin and glucolepigramin concentrations between agroecological origins, with substantially higher levels in Puno than in Junín. These results suggest that the glucosinolate profile of *Tropaeolum tuberosum* is strongly influenced by genotype and growing environment, consistent with the diversity of glucosinolate profiles reported by Ortega et al. [37]. The glucosinolate profile observed in the Junín samples resembles the sweet chemotype described by these authors, whereas the Puno samples exhibited characteristics more similar to the bitter chemotype. The Puno material originated from the Altiplano, where environmental conditions such as higher UV-B exposure may contribute to greater accumulation of aromatic glucosinolates, as discussed by Vera et al. [34].

Despite the marked reduction in glucosinalbin and glucolepigramin after processing, trace amounts of these compounds remained detectable in some processed Puno samples, whereas both compounds were absent in all processed Junín samples. This behaviour may be associated with the substantially higher initial concentrations present in the Puno material. Furthermore, glucoaubrietin was the predominant glucosinolate in both agroecological zones and was largely responsible for the increase in total glucosinolate content observed after extrusion and drum drying.

Overall, these results indicate that both agroecological origin and processing technology play important roles in determining the glucosinolate profile of instant black mashua flour. These findings are consistent with previous reports highlighting glucosinolates as key bioactive compounds contributing to the nutritional and functional value of mashua [34,37].

### 3.3. Technofunctional Properties

Table 3 presents the technofunctional properties of instant black mashua flour cultivated in the agroecological zones of Junín and Puno and obtained by extrusion and drum drying. These flours exhibited a bulk density ranging from 0.485 ± 0.003 to 0.563 ± 0.008 g/cm^3^, a water absorption index ranging from 3.72 ± 0.01 to 4.55 ± 0.06 g/g, a water solubility index ranging from 11.56 ± 0.16 to 19.22 ± 0.54%, a swelling power ranging from 4.44 ± 0.03 to 5.08 ± 0.01 g/g, and a degree of gelatinisation ranging from 97.30 ± 0.02 to 98.81 ± 0.01%.

The bulk density of instant black mashua flour obtained by extrusion was higher than that of flour produced by drum drying. This behaviour is consistent with previous studies reporting that bulk density is influenced by processing variables such as screw speed, temperature, and feed moisture content [29]. Bulk density is considered an important parameter for characterising the physical properties of extruded products and may be affected by the extent of starch transformation during processing [24].

The water absorption index (WAI) of the instant black mashua flour produced through extrusion was higher than that of the flour obtained by drum drying, indicating a greater capacity to absorb and retain water. WAI is closely associated with the disruption of starch granules and the exposure of hydrophilic groups during thermomechanical processing, serving as an indicator of polymer modification [26]. Similarly, the higher swelling power observed in extruded samples suggests a greater ability of starch to interact with water and expand. These properties may be advantageous in applications requiring rapid hydration and viscosity development, such as instant food formulations.

In contrast, drum-dried flours exhibited lower WAI and swelling power but higher water solubility index (WSI) values, particularly in the Puno samples. WSI reflects the amount of soluble material released from starch granules during processing and is commonly used as an indicator of starch degradation and molecular solubilisation. The higher WSI values observed in drum-dried flours may be related to the hydrothermal pre-cooking step (90 °C for 15 min), which likely promoted starch gelatinisation and granular disruption, increasing the release of soluble fractions. By contrast, the rapid high-temperature short-time conditions of extrusion may limit extensive molecular solubilisation, resulting in lower WSI values [20].

The degree of gelatinisation was high in all samples (>97%), indicating that both processing technologies promoted extensive starch transformation. However, drum-dried flours exhibited slightly higher gelatinisation values than extruded flours, which may be attributed to the prolonged exposure to heat and moisture during pre-cooking and drying.

Overall, the results demonstrate that processing technology significantly influences the technofunctional properties of instant black mashua flour. Extrusion promoted higher water absorption and swelling capacity, whereas drum drying favoured solubility and gelatinisation. These differences may be important when selecting the most appropriate processing method according to the intended food application.

## 4. Conclusions

The processing method used to obtain instant black mashua flour significantly affected the physicochemical, bioactive, and technofunctional properties of samples from Junín and Puno. Processing reduced moisture content and modified protein and titratable acidity, while fat, ash, fibre, and carbohydrate contents showed only minor changes relative to the fresh material. The glucosinolate profile was also altered, with changes in glucosinalbin, glucolepigramin, glucotropaeolin, and glucoaubrietin contents depending on the agroecological zone and processing method.

The effects of processing on bioactive compounds were highly dependent on the compound evaluated. Anthocyanins, vitamin C, and antioxidant capacity generally decreased after processing, whereas total phenolic and flavonoid contents increased. Carotenoid content showed a processing-dependent response, increasing after extrusion but decreasing after drum drying. Regarding technofunctional properties, extrusion generally resulted in higher bulk density, water absorption index, and swelling power, whereas drum drying produced higher water solubility and degree of gelatinisation.

Overall, these results demonstrate that both agroecological origin and processing technology significantly influence the nutritional, bioactive, and functional characteristics of instant black mashua flour. Based on the findings of this study, extrusion may be considered the most suitable processing option when the objective is to maximise glucosinolate retention, increase total phenolic and flavonoid contents, improve carotenoid stability, and obtain higher water absorption index and swelling power, properties that are desirable in instant soups, porridges, and other functional food formulations. In contrast, drum drying may be advantageous when higher water solubility and degree of gelatinisation are required, such as in beverage powders or instant drink mixes. Samples from Puno consistently exhibited higher glucosinolate contents than those from Junín, suggesting that this agroecological zone may be particularly suitable for the production of glucosinolate-rich flour. These findings provide a practical basis for selecting both raw material origin and processing technology according to the intended food application, supporting the valorisation of black mashua as a functional ingredient for the development of value-added Andean food products.

## Figures and Tables

**Table 1:** Physicochemical characterisation of instant black mashua flour obtained by different processing techniques from two agroecological areas.

Component	Agro-Ecological Areas	Instant Mashua Flour Obtained by Different Techniques		
		Fresh	Extruded	Dried
Moisture (g/100 g FM)	Junín	71.70 ± 0.65 ^c^	10.43 ± 0.04 ^b^	7.35 ± 0.03 ^a^
	Puno	68.90 ± 2.97 ^b^	9.70 ± 0.04 ^a^	7.18 ± 0.01 ^a^
Protein (g/100 g DW)	Junín	9.97 ± 0.38 ^a^	12.28 ± 0.04 ^c^	10.97 ± 0.04 ^b^
	Puno	9.41 ± 0.04 ^a^	10.22 ± 0.03 ^a^	8.94 ± 0.03 ^a^
Fat (g/100 g DW)	Junín	2.65 ± 0.22 ^a^	2.62 ± 0.02 ^a^	2.57 ± 0.01 ^a^
	Puno	2.59 ± 0.01 ^a^	2.56 ± 0.02 ^a^	2.54 ± 0.04 ^a^
Ash (g/100 g DW)	Junín	2.34 ± 0.35 ^a^	2.24 ± 0.03 ^a^	2.31 ± 0.03 ^a^
	Puno	2.25 ± 0.04 ^a^	2.29 ± 0.03 ^a^	2.19 ± 0.02 ^a^
Fibre (g/100 g DW)	Junín	3.43 ± 0.28 ^a^	3.41 ± 0.06 ^a^	2.85 ± 0.71 ^a^
	Puno	4.01 ± 0.01 ^a^	4.01 ± 0.01 ^a^	3.94 ± 0.03 ^a^
Carbohydrate (g/100 g DW)	Junín	81.61 ± 1.23 ^a^	79.45 ± 0.14 ^a^	81.31 ± 0.82 ^a^
	Puno	81.75 ± 3.08 ^a^	80.92 ± 0.13 ^a^	82.38 ± 0.14 ^a^
pH (g/100 g FM)	Junín	5.21 ± 0.01 ^a^	5.44 ± 0.01 ^a^	5.45 ± 0.01 ^a^
	Puno	5.45 ± 0.01 ^a^	4.56 ± 0.01 ^a^	5.21 ± 0.01 ^a^
Titratable acidity (g/100 g DW)	Junín	0.04 ± 0.01 ^b^	0.01 ± 0.00 ^a^	0.01 ± 0.00 ^a^
	Puno	0.09 ± 0.00 ^b^	0.01 ± 0.00 ^a^	0.01 ± 0.00 ^a^

Values are expressed as mean ± standard deviation (*n* = 3). Different lowercase letters within the same agroecological zone and component indicate significant differences among processing treatments according to Tukey’s multiple comparison test (*p* < 0.05). FM = fresh matter; DW = dry weight.

**Table 2:** Effect of processing method on the glucosinolate content of instant black mashua flour from different agroecological zones.

Component	Agro-Ecological Areas	Instant Mashua Flour Obtained by Different Process		
		Fresh	Extruded	Rotary Drum Drying
Glucosinalbin (*p*-hydroxybenzyl glucosinolate) (mg/100 g DW)	Junín	0.60 ± 0.02 ^b^	0.00 ± 0.00 ^a^	0.00 ± 0.00 ^a^
	Puno	54.19 ± 11.00 ^b^	0.35 ± 0.03 ^a^	0.00 ± 0.00 ^a^
Glucolepigramin (*m*-hydroxybenzyl glucosinolate) (mg/100 g DW)	Junín	0.64 ± 0.02 ^b^	0.00 ± 0.00 ^a^	0.00 ± 0.00 ^a^
	Puno	57.88 ± 8.11 ^b^	0.00 ± 0.00 ^a^	1.06 ± 0.01 ^a^
Glucotropaeolin (benzyl glucosinolate) (mg/100 g DW)	Junín	204.57 ± 7.20 ^c^	134.76 ± 2.12 ^b^	52.32 ± 2.86 ^a^
	Puno	45.81 ± 10.47 ^c^	80.34 ± 3.32 ^b^	273.64 ± 2.99 ^a^
Glucoaubrietin (4-methoxybenzyl glucosinolate) (mg/100 g DW)	Junín	630.51 ± 26.14 ^b^	1111.98 ± 4.22 ^c^	564.03 ± 2.68 ^a^
	Puno	976.06 ± 104.37 ^a^	1494.79 ± 8.22 ^b^	1690.39 ± 8.11 ^c^
Total glucosinolates (mg/100 g DW)	Junín	836.32 ± 33.38 ^b^	1246.74 ± 6.34 ^c^	616.35 ± 5.54 ^a^
	Puno	1135.32 ± 133.95 ^a^	1575.48 ± 11.58 ^b^	1965.08 ± 22.22 ^c^

Values are expressed as mean ± standard deviation (*n* = 3). Different lowercase letters within the same row indicate significant differences among processing treatments according to Tukey’s multiple-comparisons test (*p* < 0.05). DW = dry weight.

**Table 3:** Effect of the instant flour production process on the technofunctional properties of black mashua from different agro-ecological zones.

Agroecological Areas Zones	Processing Method	Bulk Density (g/cm^3^)	WAI (g/g)	WSI (%)	Swelling Power (g/g)	Gelatinisation Index
Junín	Extruded	0.563 ± 0.008 ^b^	4.17 ± 0.05 ^b^	16.04 ± 0.88 ^b^	4.83 ± 0.01 ^b^	97.42 ± 0.01 ^b^
	Dried	0.506 ± 0.006 ^a^	3.84 ± 0.03 ^a^	16.94 ± 0.27 ^b^	4.49 ± 0.04 ^a^	98.70 ± 0.02 ^a^
Puno	Extruded	0.552 ± 0.011 ^b^	4.55 ± 0.06 ^c^	11.56 ± 0.16 ^a^	5.08 ± 0.01 ^c^	97.30 ± 0.02 ^b^
	Dried	0.485 ± 0.003 ^a^	3.72 ± 0.01 ^a^	19.22 ± 0.54 ^c^	4.44 ± 0.03 ^a^	98.81 ± 0.01 ^a^

Values are expressed as mean ± standard deviation (*n* = 3). Different lowercase letters indicate significant differences between processing methods according to Tukey’s multiple-comparisons test (*p* < 0.05). WAI = water absorption index; WSI = water solubility index.

## Data Availability

The original contributions presented in the study are included in the article; further inquiries can be directed to the corresponding author.
